# 

**Published:** 1895-11

**Authors:** 


### CONTENTS FOR NOVEMBER, 1895.

ORIGINAL COMMUNICATIONS. PAGE.

The Law Compelling the Teaching of Physiology and Hygiene in the Public Schools. By H. R. HOPKINS, M. D. .... 289
Methods of Employing Electricity in Nervous Diseases. By FREDERICK PETERSON, M. D. 300
Reflex Epilepsy. By WILLIAM C. KRAUSS, M. D. .... 307
The Criminal's Plea of Irresponsibility. By JAMES W. PUTNAM, M. D .... 316
Nosocomial Hygiene. By STEPHEN YATES HOWELL, M. A., M. D., M. R. C. S., Eng .... 320
The Waters of Contrexéville as a Cure for Certain Arthritic Conditions. By DR. DEBOUT D'ESTREES .... 326

TRANSLATION.

A Report on Some Experiments with Trional .... 328

CLINICAL REPORT.

The Treatment of Intermittent Fever .... 333

PROGRESS IN MEDICAL SCIENCE.

Ophthalmology .... 336
State Medical Examinations .... 342
Public Health, Hygiene and Bacteriology .... 346

EDITORIAL.

Woman and the Bicycle .... 348
Topics of the Month .... 350

Personal .... 352
Obituary .... 353
Society Meetings .... 356
Reviews .... 359
Miscellany .... 364
Literary Notes .... 367

